# Familial Hypercholesterolemia: The Lipids or the Genes?

**DOI:** 10.1186/1743-7075-8-23

**Published:** 2011-04-22

**Authors:** Akl C Fahed, Georges M Nemer

**Affiliations:** 1 Department of Biochemistry, American University of Beirut, Bliss Street, Beirut, P.O. Box 11-0236, Lebanon

## Abstract

Familial Hypercholesterolemia (FH) is a common cause of premature cardiovascular disease and is often undiagnosed in young people. Although the disease is diagnosed clinically by high LDL cholesterol levels and family history, to date there are no single internationally accepted criteria for the diagnosis of FH. Several genes have been shown to be involved in FH; yet determining the implications of the different mutations on the phenotype remains a hard task. The polygenetic nature of FH is being enhanced by the discovery of new genes that serve as modifiers. Nevertheless, the picture is still unclear and many unknown genes contributing to the phenotype are most likely involved. Because of this evolving polygenetic nature, the diagnosis of FH by genetic testing is hampered by its cost and effectiveness.

In this review, we reconsider the clinical versus genetic nomenclature of FH in the literature. After we describe each of the genetic causes of FH, we summarize the known correlation with phenotypic measures so far for each genetic defect. We then discuss studies from different populations on the genetic and clinical diagnoses of FH to draw helpful conclusions on cost-effectiveness and suggestions for diagnosis.

## Introduction

Familial Hypercholesterolemia (FH) (MIM #143890) is a genetic disease characterized by elevated LDL-Cholesterol (LDL-C), which deposits in the tissues causing the external manifestations of the disease, namely tendinous xanthomas, xanthelasmas, and corneal arcus. More importantly, LDL-C deposits in blood vessels leading to premature cardiovascular disease [1,2]. The patterns of inheritance of FH were first described by Khachadurian in Lebanon before the genes that contribute to the disease were known [3]. FH was defined as an autosomal dominant disease, with a clinical distinction based on phenotype severity of a "heterozygous" and a "homozygous" form, with serum LDL-C levels that are two times and four times the normal respectively [3]. The prevalence of the severe phenotype has been reported as 1 in a million in the general population, compared to the much more common mild form with a prevalence of 1 in 500 [1]. The prevalence has been reported to be ten times higher in certain populations with a presumed founder effect, such as the Lebanese, the French Canadians, and the South Afrikaners [1,2]. A less common autosomal recessive pattern of inheritance was also described in some of the initial Lebanese families [3].

In 1986, the LDL receptor (LDLR) was discovered as the cause of Autosomal Dominant Hypercholesterolemia (ADH) [4]. It manifests a gene dosage effect such that the heterozygous and homozygous forms cause mild and severe phenotypes respectively. For years, ADH was thought of as a monogenetic disease. However, as more genotyping of FH patients was carried, patients with the phenotype but no *LDLR *mutation were discovered, and the search for other genes yielded the discovery of the Apolipoprotein B gene (*ApoB*) in 1987 [5], and the Proprotein Convertase Subtilin/Kexin 9 gene (*PCSK9*) in 2003 [6], as candidate genes in ADH. The Autosomal Recessive Hypercholesterolemia gene (*ARH*) was also discovered in 2001 [7]. These discoveries together fostered the idea of a polygenetic nature of FH.

Clinically, the severe phenotype is rarely missed with LDL-C levels that are four times higher than the normal and external manifestations since early childhood [3]. Additionally, family history is often informative of similar cases. The clinical diagnosis of the mild phenotype is much more challenging with external manifestations that might be absent or appear only in adulthood. LDL-C could also vary between upper normal levels to double the normal levels. Family history might not always be revealing. Early diagnosis of FH is crucial because the disease can be treated with lipid lowering therapy and lifestyle changes early on to prevent complications [1]. Failure to diagnose and treat FH leads to increased morbidity and mortality from premature cardiovascular disease [1,8,9].

Currently, FH can be diagnosed either clinically or genetically. The use of genetic terminologies to describe phenotypic presentations of the disease creates confusion in the literature. In this review, we set up a standard terminology for clinical and genetic descriptions of FH. We then describe the different molecular mechanisms that lead to FH and the known genotype-phenotype correlations. We finish by discussing the clinical versus genetic diagnosis of FH and by looking into worldwide models of genetic diagnosis and their mutation detection rates.

### Terminology Used to Describe Familial Hypercholesterolemia

Clinicians still use the terms "homozygous" and "heterozygous" to describe a phenotypic presentation of FH. In Lebanon, severely affected patients are labeled as 'homozygous" based on clinical assessment and are referred for LDL apheresis therapy. Screening this population recently, we have shown that less than half of them are true homozygous for an *LDLR *mutation. The rest were either combined heterozygous for two different mutations, were heterozygous for one mutation, or had no detectable mutation [10]. Only few countries currently have national genetic screening programs for FH. Cholesterol levels together with other clinical indicators remain the most used method to diagnose familial hypercholesterolemia. In table 1, we suggest a distinction in the clinical versus genetic nomenclature of FH based on whether the phenotype or the genotype is being used for diagnosis.

**Table 1:** Distinction in the Clinical Versus Genetic Nomenclature of Familial Hypercholesterolemia

Genetically (Genotype)	
Homozygous	Homozygous for a mutation in one of the candidate genes^a ^known to cause FH

Combined Heterozygous	Heterozygous for two different mutations in the same or different candidate genes known to cause FH

Heterozygous	Heterozygous for a mutation in one of the candidate genes known to cause FH

Unknown	No causative mutation could be detected after screening all candidate genes known to cause FH

**Clinically (Phenotype)**	

Severe	LDL-C levels that are three to four times the normal and external^b ^or cardiovascular^c ^manifestations of FH

Mild	Elevated LDL-C levels that do not exceed three times the normal

Pardoxical	LDL-C levels that are three to four times the normal and with no external or cardiovascular manifestations of FH OR Normal to slightly elevated LDL-C levels with external or cardiovascular manifestations of FH

^a ^Candidate genes include *LDLR*, *ApoB*, *PCSK9*, and *ARH/LDLRAP1*

^b ^External manifestations means one or more of tendinous xanthomas, xanthelasmas, or corneal arcus

^c ^Cardiovascular manifestations means the presence of premature cardiovascular disease as judged clinically

For familial clustering of cases of elevated cholesterol levels, a clinical or a genetic assessment is done. A clinical assessment of the phenotype is difficult to categorize. It is inaccurate for the most of the cases since lipid levels represent a spectrum and since many non-genetic factors can affect lipid levels and disease manifestations. To simplify, we classify the clinical nomenclature into severe, mild, and paradoxical (Table 1). While mild and severe represent two clear ends of the spectrum, paradoxical cases are those that have a more confusing presentation. A genetic nomenclature on the other hand should be used only when genotyping of the four candidate genes has been made. Heterozygous, homozygous, or combined heterozygous mutations can thus be identified (Table 1). When no mutation is detected in a mild or severe clinically diagnosed FH case, the genetic cause is unknown. When no mutation is identified in a paradoxical case, non-familial hypercholesterolemia should be considered.

### Molecular Pathways of Familial Hypercholesterolemia

#### The Molecular Pathway for the Uptake and Degradation of LDL-C by the Cell

**Figure 1:**
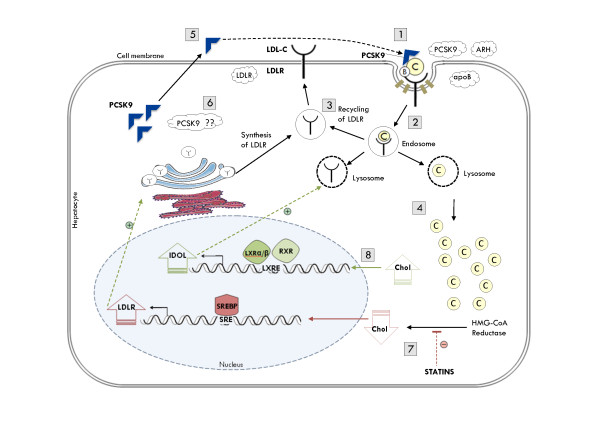
**Molecular Pathways of Disease in Familial Hypercholesterolemia **(1) The LDL receptor on the surface of hepatocytes binds ApoB-100 of the LDL particle forming a complex. (2) A clathrin-coated pit is formed and the ligand-receptor complex is endocytosed via interactions involving the LDLR Adaptor Protein 1 (LDLRAP1). (3) Inside the hepatocyte, the complex dissociates, the LDLR recycles to the cell membrane, (4) and free cholesterol is used inside the cell. (5) PCSK9 serves as a post-transcriptional inhibitor of LDLR. It is secreted and inhibits LDLR through cell-surface interactions. (6) The presence of an intracellular pathway for PCSK9-mediated LDLR inhibition is still a subject of controversy. (7) In response to decreased cholesterol such as during treatment with statins, Steroid Response Element Binding Protein (SREBP) binds to the Steroid Response Element (SRE) on the DNA and induces the transcription of the *LDLR*. (8) The sterol-responsive nuclear receptor LXR on the other hand responds to increased intracellular cholesterol inducing the transcription of IDOL, a recently discovered molecule that induces the ubiquitin-mediated degradation of the LDLR. Clouds in the figure refer to genes in which mutations have been associated with increased LDL-C levels.

The pathway was first described by Brown and Goldstein in 1986 [4]. LDL in the blood has Apolipoprotein B-100 (ApoB-100) on its surface. The LDL receptor (LDLR) is a glycoprotein found on the surface of hepatocytes and binds ApoB-100 of the LDL-C. A clathrin-coated pit is formed and both receptor and LDL-C ligand are taken into an endosome with other proteins via interactions involving the LDLR adaptor protein 1 (LDLRAP1). After dissociation of the ligand-receptor complex, LDLR is recycled to the cell membrane, while free cholesterol is used inside the cell. PCSK9 serves as a post-transcriptional LDLR inhibitor. It is secreted outside the cell and inhibits LDLR through cell surface interactions. Evidence also suggests an intracellular pathway of PCSK9-mediated LDLR inhibition, however the exact mechanism is yet to be elucidated [11]. Nuclear regulation of LDLR production includes two pathways. First, the binding of a Steroid Response Element Binding Protein (SREBP) to a Steroid Response Element (SRE) on the DNA stimulates the transcription of the *LDLR *in response to decreased intracellular cholesterol [11]. This pathway is activated during treatment with HMG-CoA Reductase inhibitors. The second player in LDLR regulation is another sterol-mediated nuclear receptor LXR, which was recently shown to induce the transcription of IDOL (Inducible Degrader of the LDLR). As its name implies, IDOL triggers ubiquitinization of the LDLR targeting it for degradation [12]. (Figure 1) This pathway ensures proper uptake of LDL-C from the blood. Any defect in this pathway results in improper uptake and high LDL-C in the blood leading to the clinical manifestations of FH.

#### LDLR

A mutation in *LDLR *(MIM#s 606945, 143890) is by far the most common cause of ADH. Null alleles produce no LDL receptors. Other alleles produce defective LDL receptors. A defective LDLR does not localize to the nuclear membrane, does not properly bind the LDL-C particle, or fails to internalize into the cell after binding [4]. The *LDLR *gene is located on 19p13 and is 45 kb long [13]. It is composed of 18 exons that code for an 860 amino acid long peptide. The LDLR protein has different domains including a signal peptide, a ligand-binding domain, an epidermal growth factor-precursor like domain, as well as O-linked sugars, transmembrane, and cytoplasmic domains [14,15]. Mutations are widely distributed along all domains of the LDLR protein and hence can result in different types of dysfunction. (Table 2) Since the discovery of the LDLR in the mid 1980s, the number of mutations has been continuously increasing. Currently, the University College of London database for the *LDLR *sequence variants lists more than 1700 hits [16,17]. Among them are nonsense substitutions or large deletions that result in absent or truncated LDLR, missense mutations that result in dysfunctional receptor, or silent mutations and other polymorphisms that do not significantly affect the function of the receptor. Many *LDLR *mutations are population specific, and many populations have a number of mutations that leads to the phenotype. In 1987, a nonsense mutation in exon 14 of the LDLR leading to a truncated receptor was discovered in Lebanese families and named the "Lebanese allele" [18]. This allele has always been associated with the Christian-Lebanese and people with Arab ancestry in the West [19,20]. Not until recently did our team study *LDLR *mutations in Lebanon and show that the Lebanese allele accounts to no more than 45% of the clinically homozygous FH patients [10]. A recent study from Tunisia shows that only 5 *LDLR *mutations are specific for the population with one of them accounting to 29.67% of cases [21]. Another example comes from Quebec where more than 90% of the heterozygous FH patients have one of eleven *LDLR *mutations [22].

**Table 2:** Gene defects involved in FH and their effect on the phenotype

Gene	Exon	Number of Sequence Variants	Function/Protein Domain	Effect on the Phenotype
*LDLR*	1	79	Signal sequence to the ER	
		
	2	82	LDL-binding domain	
			
	3	125		
			
	4	339		Gene dosage effect
			
	5	71		
			
	6	91		Homozygous → severe, resistant to therapy; death
				at early age; requires LDL apheresis
	7	105	EGF-precursor like domain	
			
	8	106		
			
	9	145		Heterozygous → variable; depends on mutation.
				Can range from normal to double the normal
	10	110		
			
	11	77		
				Known to be the most severe phenotype in general
	12	96		among all other genetic causes of FH
			
	13	72		
			
	14	100		
				Phenotype depends on modifier genes,
	15	41	OLS	environmental, and other metabolic factors.
		
	16	38	Transmembrane	
			
	17	60		
			Cytoplasmic	
			
	18	4		

*ApoB*	26	3*	Binding region to the LDLR	Less severe phenotype than LDLR mutations

*PCSK9*	1	32		
			
	2	17	Enhanced binding to LDLR	Gain of function mutations cause
				hypercholesterolemia
	3	5		
				Loss of function mutations cause
	4	14		hypocholesterolemia
			
	5	22		Polymorphisms in PCSK9 can affect the phenotype
				of FH patients in different populations (modifier gene)
	6	4		
			
	7	7		
			
	8	12		
			
	9	18		
			
	10	9		
			
	11	5		
			
	12	16		

*LDLRAP1/ARH*	1	14		
			
	2	1		Can be similar to classical homozygous FH, but has
				been reported to be less severe in general
	3	1		
			Phosphotyrosine-binding	More variable phenotype
	4	6	(PTB) domain, which is the	
			functional domain	
	5	2	responsible for cholesterol	
			metabolism	
	6	8		More responsive to lipid-lowering therapy
			
	7	6		
			
	8	1		

*Many sequence variants exist in the *ApoB *gene. Only sequence variants involved in FH are mentioned here.

#### Apo B-100

Apolipoprotein B-100 (ApoB-100) is a protein component of the LDL particle. It is found on 2p24-p23. The gene is made up of 29 exons spanning ~43 Kb and encoding two main isoforms, ApoB-48 and ApoB-100. In Familial Defective Apolipoprotein B (MIM #s 107730, 144010), LDL-C fails to bind to its ligand and remains high in the circulation [5]. There is a limited number of mutations in ApoB-100 that can cause the FH phenotype. The Arg3500Gln variant is the most famous [23]. It is common in Europe accounting to 2-5% of the FH phenotype [24]. Another variant at the same position (Arg3500Trp) is common in the Chinese population [25]. As a cause of ADH, ApoB-100 is relatively uncommon compared to *LDLR *mutations. (Table 2)

#### PCSK9

The Proprotein Convertase Subtilin/Kexin Type 9 gene (*PCSK9*; MIM# 607786) spanning 3.6 Kb on 1p32 emerged as a third locus involved in ADH, with the discovery in 2003 of two disease-causing mutations in the French population [6]. The gene spans ~25 Kb, and the 695 aa protein is encoded by twelve exons. *PCSK9 *binds to the Epidermal Growth Factor-Like Repeat A (EGF-A) domain of the LDLR inducing its degradation. Reduced LDLR levels could thus lead to hypercholesterolemia. Over the past seven years, *PCSK9 *has been heavily investigated in many populations with FH, and the databases currently list 161 sequence variants distributed along all twelve exons of the gene [14,15]. (Table 2) *PCSK9 *mutations can affect the phenotype in different ways. Gain of function mutations are rare and are associated with decreased LDLR on the surface and a severe phenotype of FH [6]. Loss of function mutations on the other hand are associated with decreased cholesterol levels [26]. Moreover, many SNPs exist in *PCSK9 *and affect cholesterol regulation differently in different populations. As a cause of ADH, *PCSK9 *is rare compared to *LDLR *and *ApoB-100*; however, large numbers of *PCSK9 *polymorphisms are associated with cholesterol levels in population studies [27]. Recent studies are focusing on the potential of PCSK9-inhibiting compounds as a therapeutic target for dyslipidemias [28-30].

#### ARH

Since the initial observations on the mode of inheritance of FH, an autosomal recessive pattern has been noted [3]. In 2001, Autosomal Recessive Hypercholesterolemia (ARH) was found to be caused by mutations in the LDL Receptor Adaptor Protein 1 (*LDLRAP1*) also referred to as the *ARH *gene [7]. The gene is mapped to 1p36-35 [31] spanning ~25 Kb with 9 exons coding for a 308 aa protein. In ARH, the internalization of the ligand-receptor complex cannot occur and all the LDL receptors accumulate on the cell membrane. ARH is extremely rare compared to ADH, and the number of patients described to have defects in the *ARH *gene does not exceed 100 [32]. ARH was initially described in Sardinian and Lebanese families, but later found in American, Iranian, Japanese, Mexican, Asian, Indian, English, Turkish, and Syrian families [32-34].

### Genotype Phenotype Correlations

*LDLR *mutations show a gene dosage effect, and a classical presentation of homozygous versus heterozygous FH patients has been documented. However, with the advances in sequencing strategies it became clear that *LDLR *mutations did not describe it all. Many patients with severe or moderate phenotypes did not carry any *LDLR *mutation. Later studies showed Familial Defective *ApoB *[5], *ARH *[7], and more recently *PCSK9 *[6] as possible explanations for an *LDLR *defect-negative FH phenotype.

#### LDLR

Table 2 shows phenotype comparisons between the four different genes involved in FH. In general, the classical ADH patients with *LDLR *mutations have the worst phenotype with the highest lipid levels and the least response to lipid-lowering therapy. Homozygotes usually necessitate LDL apheresis therapy otherwise they die of cardiovascular events as young as adolescence. Heterozygotes have moderately elevated lipid levels, external manifestations by adulthood or not at all, and premature cardiovascular disease [1,8,9].

#### ApoB-100

*ApoB-100 *mutations show incomplete penetrance, so patients with Familial Ligand-Defective Apolipoprotein B show in general a less severe phenotype than FH patients with *LDLR *mutations [24]. Still in many instances however, heterozygous *ApoB *defective patients can be clinically indistinguishable from heterozygous *LDLR *mutation patients. It was estimated that at least 2-5% of FH patients in lipid clinics are due to *ApoB-100 *mutations [24]. Considering *ApoB-100 *mutations is particularly important in populations where it is known to be common, namely European and North American [24], and less important in populations where it is rarely reported such as Arabs and Middle Easterns [35].

#### ARH

*ARH *also shows some phenotypic differences from the classical *LDLR *mutants. Patients have lower lipid levels, traditionally observed to be somewhere between the levels seen in heterozygous and homozygous ADH patients. However, this does not always hold true, and there seems to be a great variability of the phenotype between patients in ARH, even within the same family [36]. A report of LDL kinetic studies on one patient with Turkish decent harboring an *ARH *mutation showed that the LDL catabolic rate was delayed up to three-fold, making the patient indistinguishable from patients with homozygous *LDLR *mutations [37]. In general, ARH patients show a better response to lipid-lowering therapy than the ADH patients, and they rarely require LDL apheresis [38]. Some studies also reported increased HDL levels in ARH compared to ADH. The incidence of cardiovascular events in ARH also tends to be delayed and they rarely have any in adolescence [36]. Most importantly in FH is the family history. *LDLRAP1 *mutations should always be suspected in patients who are products of consanguineous marriages, in typical populations, and with an autosomal recessive pattern of inheritance.

#### PCSK9

The discovery of *PCSK9 *has added a lot to the phenotypic understanding of FH. We have established earlier that gain of function mutations in this gene cause hypercholesterolemia and loss of function mutations cause hypocholesterolemia, and that the gene is greatly polymorphic with population differences. This has established *PCSK9 *as a modifier gene in FH, which causes the significant phenotypic variability even in patients carrying the same *LDLR *mutation [39]. Many studies have looked at the presence of *PCSK9 *sequence variants on top of *LDLR *mutations [39-41]. For some combined mutants, the phenotype is as severe as that of homozygous *LDLR *mutants [27].

#### The Diagnostic Gap in FH

Still many clinically diagnosed FH patients fail to show any mutation in these four genes. This diagnostic gap is observed in most clinically diagnosed FH cohorts who are screened for mutations. Canadians have studied this diagnostic gap in Ontario and showed that exon-by-exon sequencing analysis (EBESA) diagnosed only two thirds the FH patients [42]. Using the Multiplex Ligation-Dependent Probe Amplification (MLPA) technique to detect copy number variations (CNVs) [43], they could detect an abnormality in two thirds of the remaining gap, reducing it from 30% to 10% [42]. Their findings suggested that heterozygous *LDLR *CNV's are associated with more severe phenotypes and they are usually missed in EBESA [22]. Another major explanation of the diagnostic gap is the presence of mutations in other unknown novel genes that are involved in cholesterol metabolism. More mapping studies to look for novel genes involved in FH are needed to fill the diagnostic gap.

#### Variability of the Phenotype

FH is a disease that shows great phenotypic variability [44]. The polygenetic nature of the disease is being enhanced with the discovery of more modifier genes, which explains a large part of this phenotypic variability. In our cohort of Lebanese FH patients, we identified many heterozygotes for the Lebanese allele mutation in the *LDLR*, yet having normal lipid levels on no therapy [10]. So far we have been referring to the phenotype of FH patients in terms of lipid levels only. However, other phenotypic measures in this population include onset of hypercholesterolemia, onset and degree of atherosclerosis, cardiovascular measurements such as aortic stenosis, carotid plaques, and intima-media thickness, cardiovascular morbidity and mortality, and response to lipid-lowering therapy among others. All these phenotypic measures are the result of not only lipid levels, but also a combination of genetic, metabolic, and environmental factors. People carrying the same mutation can have different lipid levels, and certain populations have moderate phenotypic expression of apparently severe mutations [21,45]. The type of *LDLR *mutation has been shown to correlate with the response to statin therapy [46]. Polymorphisms in lipid modifier genes, such as apolipoproteins, particularly ApoE, can significantly affect the FH phenotype [47]. Conventional risk factors for atherosclerosis such as smoking, diet, hypertension, and diabetes are also additive in FH [48,49]. The levels of lipoprotein (a) have been correlated with atherosclerosis and could also explain a variable phenotype or a paradoxical case of FH [50].

### The Clinical Diagnosis of Familial Hypercholesterolemia

#### The Three Sets of Clinical Criteria for the Diagnosis of FH

Early diagnosis of heterozygous FH allows for prompt treatment and prevention of morbidity and mortality from premature cardiovascular disease. Tremendous efforts have been made to improve the early diagnosis of this population, yet, there is no single internationally accepted set of criteria for the clinical diagnosis of FH. There are three sets of statistically and genetically validated criteria however that are most commonly used: the Dutch [51], the UK [52], and the US [53] criteria. (Table 3) The US MEDPED developed two sets of criteria distinguishing between the general population and close relatives of known FH patients. Criteria differ in each group due to the statistical component of a pre-determined probability. The statistical criteria developed are based solely on lipid levels and age, and they are highly sensitive and specific [53]. (Table 3) The Simon Broome Register Group in the UK as well as the MEDPED group in the Netherlands developed their criteria by classifying definite, probable, and possible diagnoses of FH. Unlike the US criteria, which used only lipid levels, the UK and Dutch criteria use family history, personal history, and physical signs in addition to the cholesterol levels [51,52].

**Table 3:** Criteria for the Clinical Diagnosis of Familial Hypercholesterolemia

MEDPED Criteria ( USA)					
	**Total Cholesterol (LDL-C) levels in mg/dL**				**Comments**
	
**Age**	**1**^**st **^**degree** ** relative**	**2**^**nd **^**degree ** **relative**	**3**^**rd **^**degree ** **relative**	**General ** **Population**	
	
<18	220 (155)	230 (165)	240 (170)	270 (200)	
	
20	240 (170)	250 (180)	260 (185)	290 (220)	98% specificity
	
30	270 (190)	280 (200)	290 (210)	340 (240)	87% sensitivity
	
40 +	290 (205)	300 (215)	310 (225)	360 (260)	

**Simon Broome Criteria (UK)**					

Total Cholesterol (LDL-C) in mg/dL 290 (190) in adults, or 260 (155) in pediatrics	AND	DNA mutation			Definite FH
		
		Tendon xanthomas in the patient or in a 1^st ^or 2^nd ^degree relative			Probable FH
		
		Family history of MI at age <50 in 2^nd ^degree relative or at age <60 in 1^st ^degree relative OR Family history of total cholesterol >290 mg/dL in 1^st ^or 2^nd ^degree relative			Possible FH

**Dutch Criteria (The Netherlands)**					

1 point	1^st ^degree relative with premature cardiovascular disease or LDL-C >95^th ^ percentile, or Personal history of premature peripheral or cerebrovascular disease, or LDL-C between 155 and 189 mg/dL				Definite FH ( = or > 8 points)
	
2 points	1^st ^degree relative with tendinous xanthoma or corneal arcus, or 1^st ^degree relative child (<18 yrs) with LDL-C > 95^th ^percentile, or Personal history of coronary artery disease				
	
3 points	LDL-C between 190 and 249 mg/dL				Probable FH (6-7 points)
	
4 points	Presence of corneal arcus in patient less than 45 yrs old				
	
5 points	LDL-C between 250 and 329 mg/dL				Possible FH (3-5 points)
	
6 points	Presence of a tendon xanthoma				
	
8 points	LDL-C above 330 mg/dL, or Functional mutation in the *LDLR *gene				

#### Advantages and Disadvantages of Clinical Diagnosis

Although the above clinical criteria for diagnosis might be helpful in diagnosing relatives of known FH patients, they are not accurate in diagnosing index cases in the general population. They are very helpful though in avoiding the informal assessment of patients, which is very often a weak predictor of FH. The advantage of clinical criteria is also their low cost as they depend solely on history taking, physical exam, blood lipid profile testing, and possibly noninvasive cardiovascular testing. Clinical diagnosis will fail to distinguish between the classical FH due to *LDLR *mutations and the other genetic causes of FH such as *ApoB-100*, *ARH*, and *PCSK9*, or even non-familial hypercholesterolemia such as secondary hypercholesterolemia, sitosterolemia, and others. More importantly, clinical diagnosis could miss a considerable proportion of the FH patients, particularly those with a mild phenotype and the pediatric population in whom the phenotype has not appeared yet. Very often, a myocardial infarction is the first presenting sign in many FH patients. Finally, clinical diagnosis will not allow for understanding known genotype phenotype correlations such as the better response to statin therapy in *ApoB-100 *and *ARH *compared to *LDLR *mutations.

### The Genetic Diagnosis of Familial Hypercholesterolemia

#### Importance of a DNA Diagnosis

Genetic testing may give a definite diagnosis of FH if a pathological mutation were detected [54]. Early and definite diagnosis of FH has large benefits since it allows for cholesterol lowering and risk prevention [54]. DNA diagnosis is particularly important in equivocal cases where lipid levels are mild with no clear external manifestations and with a family history of premature coronary artery disease. These comprise the majority of the cases of FH. In the extreme case, a patient with an *LDLR *mutation might have LDL-C levels that fall within the normal range. We have pinpointed few of these cases in the Lebanese cohort. Although there is no evidence that suggests that the mutation by itself poses an independent risk for cardiovascular disease, identifying such a mutation is clinically important since the patient can develop high LDL-C levels at any point in life and be missed. Finding a known pathogenic mutation might prompt the clinician to screen more frequently for hypercholesterolemia. This concept is most useful in pediatrics where lipid levels might not be high enough to make a diagnosis, although genetic testing in the pediatric population remains a subject of controversy [55]. A recent Cochrane review established the efficiency and short-term safety of lipid-lowering therapy in children with FH [56]. Hence, an accurate and early diagnosis might allow for treatment early on to prevent cardiovascular disease morbidity and mortality.

Due to the paucity of data on genotype phenotype correlations, clinical diagnosis will miss a large percentage of FH patients. It is currently estimated that only 15 to 20% of patients with FH are actually diagnosed [57,58]. A study on 643 Danish probands could not even find a single phenotypic characteristic to predict the existence of a mutation [59]. A more recent study on 696 possible FH patients in Portugal showed that genetic diagnosis for cardiovascular risk stratification was superior to clinical diagnosis using the Simon Broome criteria [60]. Not only does finding a mutation allow for early diagnosis and treatment, but it also has prognostic value. Different mutations can dictate different directions of management, such as the poorer response to lipid-lowering therapy with certain *LDLR *mutations [46]. The identity of the gene involved, dictates some aspects of the phenotype as we already established in the genotype-phenotype correlations. Although still not completely understood, such correlations can potentially aid the clinician to decide on how aggressive the treatment strategy will be. The effect of the different *LDLR *mutations on the response to statins was studied in a limited number of small-scale studies in which several showed statistically significant correlations [46]. Nevertheless, such pharmacogenetic variability should be studied in large randomized control trials, which is a little bit challenging in the presence of a huge number of mutations in the *LDLR*.

Finally, the phenotypic expression of the FH mutation may skip generations. This can occur for instance due to the presence of modifier genes that can decrease LDL-C levels or due to epigenetic factors that might also modulate the phenotype. In such cases, genetic testing may have a prognostic significance for succeeding generations. For this reason, discovery of a known pathogenic mutation in an individual with normal LDL-C levels prompts the clinician to screen other family members who might have undiagnosed hypercholesterolemia.

#### Population Screening

In 1997, the WHO clearly established the benefits of a DNA test for the diagnosis of FH and re-assured that it is cost-effective [61]. However, with the evolving polygenetic nature of the disease, several studies showed that genetic diagnosis is hampered by the high cost, and genetic screening for the population at large failed to show cost-effectiveness due to the polygenetic nature of the disease [62]. Nevertheless, for certain populations where one or few known mutations cause the disease, and where the prevalence of FH is higher than the general population, population screening might be a good strategy. However, until genetic epidemiology studies are conducted on these populations, it will be hard to comment. Another limitation of genetic population screening for FH is the variability of the phenotype [44] and the paucity of data in genotype phenotype correlations. Moreover, the phenotype is affected by many non-genetic factors as mentioned earlier [47-49]. A recent meta-analysis showed a benefit for population screening of children ages 1 to 9 years using serum lipid levels and suggested that this strategy might be helpful in identifying new cases in two generations, the children and their parents [63].

#### Cascade Screening

Cascade screening is another strategy that proved to be cost-effective in genetic testing for FH. In cascade screening, an index patient is diagnosed initially clinically through one of the clinical criteria listed in Table 3. A DNA test confirms the mutation in the index patient. Screening for the same mutation is undertaken in first degree relatives to look for new cases. New confirmed cases from the relatives are treated as new index cases and their first degree relatives are screened. The first successful model of national genetic cascade screening programs came from the Netherlands, which started in 1994 [64-66]. Norway also had successful results with their program started in 2003 [67,68]. A large percentage of the relatives screened ended up having definite FH, and many of them were not on any therapy at the time of diagnosis. Other countries that are starting to follow similar strategies include Spain [69,70], Australia and New Zealand [71,72], and Wales [73,74]. Table 4 summarizes mutation detection rates in these genetic cascade screening programs as reported in the most recent literature. It also lists mutation detection rates in clinically diagnosed cohorts of patients from these countries [70,72,74,75] and others such as Denmark [76,77]. Mutation detection rates differ based on the original clinical diagnosis of the cohort and on the mutation detection method. Various mutation detection methods are used in different countries, including direct sequencing [66], arrays [70], or Denaturing High Performance Liquid Chromatography (DHPLC) and melting analysis [78,79]. Most screening strategies cover the *LDLR *and *apoB-100 *genes. An more novel screening strategy has been implemented in Iceland whereby ancestors of FH probands were traced and the oldest in each family lineage was screened for the common *LDLR *Icelandic mutation, I4T +2C [80]. This genealogical tracing might be superior to the conventional first-degree relative approach in founder populations.

**Table 4:** Mutation Detection Rates in Models of Genetic Screening for Familial Hypercholesterolemia

Country	Start Date	Years assessed	Screening	Relatives of index cases	Clinically diagnosed patients	Mutation detection rate	Mutation detection method *	Clinical diagnosis before screening	Reference
Netherlands	1994	16	Cascade	43891	-	36%	Direct sequencing of promoter and all exons of LDLR and exons 26 and 29 of *apoB*; MLPA for large deletions	N/A	[66]
	
	-	-	Patient screening	-	1465	44%	Stepwise screening approach for *LDLR *and *apoB*	The Dutch Criteria	[75]

Norway	2003	5	Cascade	1805	-	44.8%	Direct sequencing of promoter and exons 1-17 and coding part of exon 18 of the *LDLR *and of codon 3500-containing PCR fragment of the *apoB *gene; MLPA for large deletions	N/A	[67],[68]

Iceland	2003	N/A	**Systematic family screening**	**68**	-	**59%**	Screened for the common *LDLR *Icelandic mutation (I4T +2C) only	N/A	[80]

Denmark	-	-	Patient screening	-	1053	40.4%		Two out of three:	[76]
							Stepwise screening approach for *LDLR *and *apoB*	(i) Elevated LDL-C	
	1995	8	Patient screening	-	408	33.1%		(ii) Premature CAD or family history of CVD; (iii) Presence of xanthomas	[77]

Spain	2004	3	Patient screening	-	825	55.6%	Lipochip (Microarray that includes 203 *LDLR *and 4 *ApoB *mutations)	Elevated familial LDL-C with or without familial or personal histories of premature CAD or xanthomas	[69,70]

UK	2005	-	Patient screening	-	635	36.5%		Definite or probable FH	[74]
							Commercial amplification refractory mutation system (ARMS) for 18 *LDLR*, one *apoB *and one *PCSK9 *mutations		
			Cascade	296	-	56.1%		N/A	

New Zealand	2004	4	Patient screening	-	588	13%		Elevated LDL-C, lipid stigmata, or family history of premature CVD	[72,78,79]
							Denaturing High Performance Liquid Chromatography (DHPLC) and melting analysis with direct sequencing to look for mutations in *LDLR *and apoB		
			Cascade	353	-	45%		N/A	

* For countries where mutation detection methods have changed over the years, the current mutation detection method at the time of the published study is listed.

#### Implementation Issues

Although cascade testing is a successful and cost-effective model for early diagnosis and treatment of FH, its implementation carries many considerations. Currently there is no study that could genetically identify the cause of 100% of a clinically diagnosed FH population, and a large part of that is due to the polygenetic nature of the disease. This complicates DNA testing and necessitates the development of clear national guidelines that provide step-by-step criteria for screening for particular genes, based on previous genotype data on the population. Such a national system would necessitate an infrastructure to accommodate it, including education and training, specialized clinics, outreach, etc. A genetic testing program also carries with it ethical considerations, psychological implications, and insurance coverage issues [81].

### The Lipids or the Genes?

Familial Hypercholesterolemia has been historically diagnosed and described based on lipid levels and family history. LDL-C levels also were the major determinant of the phenotype. The advances in genetic testing have added a different perspective to the disease. Not only does genetic diagnosis provide a more accurate and early diagnosis of FH, but it also provides information about the phenotype and the prognosis that could not be known from lipid levels alone. It also allows for the identification of more silent cases in the population, decreasing the incidence of premature cardiovascular disease. Although proven cost-effective, the move from lipids to genes is challenging and will require huge efforts from researchers and public health systems.

## Conclusions

• Familial Hypercholesterolemia is caused by mutations in *LDLR*, *ApoB-100*, *PCSK9*, and *LDLRAP1*.

• The terms homozygous and heterozygous refer to a definite genotype of a patient with FH, while the phenotype is a variable spectrum that could potentially be described as mild, severe, or paradoxical.

• The majority of people with FH have a mild phenotype, are undiagnosed and untreated, and ultimately develop premature cardiovascular disease.

• FH is a polygenetic disease with known and unknown genes. It demonstrates a large variability in the phenotype not only due to the polygenetic nature, but also due to non-genetic factors.

• A DNA diagnosis for FH is the only definite diagnosis for the disease.

• Cascade genetic screening for FH is cost-effective and should be adopted by national healthcare programs.

## List of Abbreviations

LDL: Low Density Lipoprotein; MEDPED: Make Early Diagnosis to Prevent Early Deaths.

## Competing interests

The authors declare that they have no competing interests.

## Authors' contributions

AF and GN reviewed the literature and wrote different sections of the manuscript equally. Both authors read and approved the final manuscript.

## Authors' Information

AF is an MD and a post-doctoral research fellow in Cardiogenetics at the American University of Beirut. GN is an Associate Professor of Biochemistry and Director of the Congenital Heart Disease Genetic Program (CHDGP) at the American University of Beirut.
